# Characteristics of spirochetemic patients with a solitary erythema migrans skin lesion in Europe

**DOI:** 10.1371/journal.pone.0250198

**Published:** 2021-04-22

**Authors:** Vera Maraspin, Katarina Ogrinc, Tereza Rojko, Petra Bogovič, Eva Ružić-Sabljić, Andrej Kastrin, Gary P. Wormser, Franc Strle

**Affiliations:** 1 Department of Infectious Diseases, University Medical Center Ljubljana, Ljubljana, Slovenia; 2 Institute for Microbiology and Immunology, Faculty of Medicine, University of Ljubljana, Ljubljana, Slovenia; 3 Institute for Biostatistics and Medical Informatics, Faculty of Medicine, University of Ljubljana, Ljubljana, Slovenia; 4 Division of Infectious Diseases, New York Medical College, Valhalla, New York, United States of America; University of North Dakota School of Medicine and Health Sciences, UNITED STATES

## Abstract

Neither pre-treatment characteristics, nor the outcome after antibiotic therapy, have been reported for spirochetemic European patients with Lyme borreliosis. In the present study, patients with a solitary erythema migrans (EM) who had a positive blood culture for either *Borrelia afzelii* (n = 116) or *Borrelia garinii* (n = 37) were compared with age- and sex-matched patients who had a negative blood culture, but were culture positive for the corresponding *Borrelia* species from skin. Collectively, spirochetemic patients significantly more often recalled a tick bite at the site of the EM skin lesion, had a shorter time interval from the bite to the onset of EM, had a shorter duration of the skin lesion prior to diagnosis, and had a smaller EM skin lesion that was more often homogeneous in appearance. Similar results were found for the subset of spirochetemic patients infected with *B*. *afzelii* but not for those infected with *B*. *garinii*. However, patients with *B*. *garinii* bacteremia had faster-spreading and larger EM skin lesions, and more often reported itching at the site of the lesion than patients with *B*. *afzelii* bacteremia. Treatment failures were rare (7/306 patients, 2.3%) and were not associated with having spirochetemia or with which *Borrelia* species was causing the infection.

## Introduction

Lyme borreliosis (LB) is the most common tick-transmitted disease in the Northern hemisphere. In North America it is nearly exclusively caused by *Borrelia burgdorferi* sensu stricto (hereafter referred to as *B*. *burgdorferi*), while in both Europe and Asia the majority of patients with LB are infected with *Borrelia afzelii*, *Borrelia garinii* or *Borrelia bavariensis*. Differences in the clinical features of LB between North America and Europe are believed to be attributable to the different species causing the infections [1–3]. Nevertheless, irrespective of the causative agent, LB usually presents with a characteristic skin lesion called erythema migrans (EM). This lesion, which is the result of introduction of Lyme borrelia (*Borrelia burgdorferi* sensu lato pathogenic for humans) into the skin through a tick-bite, develops within days to a few weeks after the bite. In some patients the causative agent disseminates through the blood, resulting in secondary skin lesions (multiple EM, MEM) and/or involvement of the nervous system, joints, and/or heart [1–3]. Thus, with the exception of a solitary EM, borrelial lymphocytoma and probably also acrodermatitis chronica atrophicans, the other clinical manifestations of LB occur most likely as the result of hematogenous dissemination of borreliae from the initial skin infection site. *B*. *afzelii* has the propensity to cause skin manifestations of LB, whereas *B*. *garinii* and *B*. *bavariensis* are the main cause of Lyme neuroborreliosis in Europe. Untreated infection with *B*. *burgdorferi* strains in North America often leads to arthritis [1–3]. These clinical manifestations do not appear immediately after the onset of an EM skin lesion but instead after a time lag of a few days in patients who develop MEM, after a few weeks in patients who develop Lyme neuroborreliosis and after weeks to months in patients who develop arthritis. While these manifestations are the clinical consequences of dissemination, the gold laboratory standard for demonstration of bacteremia, which is a prerequisite for hematogenous dissemination, is cultivation of borreliae from blood. It is believed that there is a time lag between bacteremia and the seeding of borreliae in tissue, with an additional time lag until the appearance of the corresponding clinical manifestation.

Isolation of borreliae from the blood succeeds mainly in patients with EM, rather than in patients with other clinical manifestations of LB, suggesting that dissemination occurs early in the course of LB [4–9]. Detection of spirochetemia is much more common in the USA than in Europe. In adult US patients with EM, spirochetemia can be detected in 40–75% of cases, depending on the method used, while in Europe the rate of borrelia isolation from blood is only 1.2–7.7% [4, 5, 8, 9]. The low sensitivity of borrelia blood cultures is probably an important reason that data on characteristics of patients with culture-confirmed hematogenous dissemination of borreliae in Europe are limited. Indeed, according to a PubMed literature search performed in November 2020 (and using the key words “borrelia” OR “Borrelia afzelii” OR “Borrelia garinii” AND “blood”), several studies on the isolation of *B*. *afzelii* or *B*. *garinii* from skin or cerebrospinal fluid with corresponding clinical data have been published [10–18]. However, no information on the pre-treatment characteristics or the post-treatment outcome of LB for patients with isolation of *B*. *afzelii* or *B*. *garinii* from blood has been reported. In the present study we focused on patients with a solitary EM skin lesion for whom isolation of borreliae from blood was successful.

## Materials and methods

### Selection of patients

The clinical part of this study was performed at the Department of Infectious Diseases, University Medical Center (UMC) Ljubljana, Slovenia, from 1995 through 2018. Clinical data were acquired using a structured questionnaire. Information on the patients had been systematically collected in a similar way through the whole 24-year period. Fulfillment of the following inclusion criteria was required:

i) Age ≥ 15 years,
ii) Presence of a solitary EM, defined by the criteria according to Stanek et al. [19],
iii) Consent from study subjects that included obtaining a skin biopsy of the EM lesion and a blood specimen for culture of *B*. *burgdorferi* sensu lato,
iv) Identification of *B*. *afzelii* or *B*. *garinii* from a blood culture, and
v) One-year follow-up period.

### Antibiotic therapy

Most of the patients evaluated were already enrolled in different treatment trials conducted by Department of Infectious Diseases of the UMC Ljubljana. Therefore, the choice of the prescribed antibiotic was influenced by the criteria established for each separate treatment study. The majority of patients received an oral antibiotic: doxycycline– 46 patients (100 mg b.i.d.; 43 patients for 14 days, 3 patients for 10 days), cefuroxime-axetil– 34 patients (500 mg b.i.d. for 15 days), amoxicillin– 18 patients (500 mg t.i.d. for 14 days), or azithromycin– 37 patients (500 mg b.i.d. for the first day, followed by 500 mg o.d. for 4 days). Patients with more severe clinical symptoms such as headache, myalgia, arthralgia and fatigue, and pregnant women were instead treated with ceftriaxone i.v.– 18 patients (2 g o.d. for 14 days).

### Clinical evaluation

At enrollment, a complete medical history with demographic, epidemiological, and clinical data was obtained, and a physical examination was performed. Attention was focused on the presence of objective manifestations of LB, such as signs of nervous system, joint or skin involvement, the occurrence of local symptoms at the EM skin site (itching, burning, and pain), and the incidence of concomitant, newly developed or worsened systemic symptoms (fatigue, headache, myalgia, arthralgia, fever, dizziness, nausea) temporally related to the onset of the EM for which no other known medical explanation was found (LB associated symptoms). The largest and the smallest diameters (in cm) of the EM skin lesion were measured at the first clinical evaluation. To determine the speed of increase of the skin lesion, the largest diameter of EM (cm) was divided by the duration of EM skin lesion (days). Furthermore, the area of skin involvement was calculated using the formula for the surface of the ellipse in square centimeters (largest diameter × smallest diameter × π / 4). The daily increase of the surface area was determined by division of the area of the EM at the first clinical evaluation (cm^2^) by the duration of the EM skin lesion (days). Patients were re-evaluated at day 14, and at months 2, 6, and 12 after the onset of treatment of EM.

### Laboratory analyses

Laboratory analyses included an erythrocyte sedimentation rate, a complete blood cell count with differential and platelet count, and liver function tests, performed at presentation and again at the 14-day follow-up visit. Patients with abnormal laboratory findings detected at the follow-up visit 14 days after the first visit were retested 2–3 months later. For the majority of patients the abnormalities resolved but not for all. If no other explanation was found, the abnormalities were interpreted to be from LB, however, such findings were not interpreted as treatment failure.

### Serological testing

At the first visit, and at the follow-up visits at months 2, 6, and 12, serologic testing for the presence of IgM and IgG antibodies to *B*. *burgdorferi* s.l. were determined. In the period up to August 2011, an indirect immunofluorescent test (IFA) was performed, using a local isolate of *B*. *afzelii* as the antigen (titers ≥1:256 were considered positive) [20]. From September 2011 onwards, an indirect chemiluminescence immunoassay (LIAISON^®^; DiaSorin, Saluggia, Italy) with the antigens OspC and VlsE for detection of IgM antibodies and VlsE for IgG antibodies was utilized [21]. Positive results were interpreted according to the manufacturer’s instructions.

### Cultivation and identification of *Borrelia* strains

Skin biopsy and blood specimens of patients with EM were cultured. The skin biopsy was performed at the border of the EM skin lesion following disinfection with 70% alcohol and local anesthesia with 2% xylocaine. Specimens were cultivated in an in-house prepared modified Kelly Pettenkofer (MKP) medium [22] and were examined microscopically for the presence of borreliae for up to 9 weeks.

After disinfection of skin (70% alcohol), whole blood (5 ml in the period from 1992 to 2000, 9 ml from 2001 onwards) was obtained by venipuncture and placed in tubes with sodium citrate. Samples were centrifuged (500 rpm for 10 minutes), and plasma was inoculated into tubes with 7 ml of MKP (1 ml of plasma per tube). All plasma samples were incubated at 33° C and examined weekly by dark-field microscopy for the presence of spirochetes for up to 12 weeks [23].

In patients with either a positive skin or blood culture, each procedure was repeated 2–3 months later; the repeat skin biopsy was performed at the site of the first biopsy.

Skin and blood isolates were identified to species level using pulsed-field gel electrophoresis following *MluI* restriction of genomic DNA (*MluI*-restriction fragment length polymorphism, RFLP) or by PCR-based MseI-RFLP of the 5S-23S intergenic region [24, 25].

### Treatment failure

In this study treatment failure was defined as: i) development of MEM or an objective extracutaneous manifestation of LB, ii) appearance/persistence of subjective symptoms fulfilling criteria for LB associated symptoms that were severe enough to substantially interfere with normal life functioning, iii) persistence of EM for 2–3 months after the initiation of antibiotic treatment (i.e., a visible EM at a follow-up visit 2–3 months after the first examination), or iv) demonstration of borreliae in a repeat blood or skin culture 2–3 months after beginning antibiotic therapy. Patients regarded as a treatment failure were re-treated with ceftriaxone (i, ii) or with an alternative antibiotic administered orally (iii, iv).

### Selection of the control group

For each patient with a solitary EM and isolation of *B*. *afzelii* or *B*. *garinii* from blood, one sex-, age- (±2 years), and antibiotic treatment-matched patient, diagnosed with a solitary EM at the Department of Infectious Diseases, UMC Ljubljana in the same year, who had a negative borrelia blood culture result, and for whom the skin biopsy grew the same species of Lyme borrelia as the study subject, was assigned. The only miss-match was for 18 patients who received ceftriaxone, whereas the 18 control subjects were treated with doxycycline. If more than 1 control was identified fulfilling all 3 criteria (including exact age), we chose the control whose initials were the closest to the corresponding patient surname. The sex-, age-, and antibiotic treatment-matching was performed because these parameters are deemed to be associated with the course and outcome of early LB [26].

### Clinical and laboratory comparisons between LB patients’ groups

Several comparisons of the pre-treatment characteristics and the course and outcome of EM were performed for patients.

More specifically, we compared findings in:

i) patients with isolation of *B*. *afzelii* from blood vs. patients with isolation of *B*. *garinii* from blood;
ii) patients with isolation of *B*. *afzelii* from blood vs. controls with isolation of *B*. *afzelii* from the skin but not from the blood;
iii) patients with isolation of *B*. *garinii* from blood vs. controls with isolation of *B*. *garinii* from the skin but not from the blood.
iv) controls with isolation of *B*. *afzelii* from skin vs. controls with isolation of *B*. *garinii* from skin; and
v) the entire group with spirochetemia vs the entire group with isolation of borreliae only from skin.

### Statistical methods

Categorical variables were summarized with frequencies, and percentages and 95% confidence intervals (CI), and numeric variables with medians and interquartile ranges.

The characteristics of the individual groups were compared using the Mann–Whitney *U* test for numerical variables and the chi-square test with Yates’ continuity correction for categorical variables. *P* values < 0.05 were interpreted as significant.

Additional statistical analysis was carried out in two steps. First, a regression model with Least Absolute Shrinkage and Selection Operator (LASSO) was used as a variable selection method [27]. The complexity parameter λ, which determines the amount of shrinkage, was estimated using a repeated 5-fold cross-validation procedure with one-standard-error (1SE) rule. Next, all variables selected by the LASSO model (whose penalized estimates were not shrunk to 0) were used for further analysis. The association between response variable and selected covariates was estimated using multiple logistic regression. The results are presented as adjusted odds ratios (ORs) with 95% CIs. R language for statistical computing was used for all analyzes. The LASSO models were computed using the glmnet package.

### Ethics

Patients evaluated in the present study had been enrolled predominantly in different treatment studies conducted by Department of Infectious Diseases of the UMC Ljubljana that included skin and blood culturing and follow-up as presented for the present study. Each of these studies was approved by the Medical Ethics Committee of the Ministry of Health of the Republic of Slovenia (No 133/06/03, 38/05/06, 144/06/07, 35/05/09, 36/05/09, 127/06/10, 17/11/12, and 145/45/14), and for each patient written informed consent was obtained.

## Results

In the period from 1995 to 2018, borreliae had been isolated from the blood of 219 adult patients with LB; 170 of them had a solitary EM and 153 also fulfilled all of the other inclusion criteria and qualified for the present study. Of the 153 evaluable patients with spirochetemia, *B*. *afzelii* was isolated from 116 patients and *B*. *garinii* from 37 patients. For 139 patients a skin biopsy was also performed, and in 65/139 (46.8%) borreliae were isolated from skin. For all the patients the *Borrelia* species isolated from skin was the same species as the blood isolate. Controls consisted of 153 patients with a solitary EM, who had a negative borrelia blood culture, but for whom *B*. *afzelii* (n = 116) or *B*. *garinii* (n = 37) had been isolated from a skin culture.

### Pre-treatment findings

Comparison of the 153 patients with either *B*. *afzelii* or *B*. *garinii* isolated from blood with the group of 153 patients with the corresponding *Borrelia* species isolated only from skin (S1 Table) revealed that spirochetemic patients significantly more often recalled a tick bite at the site of the EM skin lesion (105/153, 68.6% versus 74/152, 48.7%; *P*<0.001), had a shorter time interval from the bite to the onset of EM (10.5 versus 19 days; *P*<0.001), had a shorter duration of the skin lesion prior to diagnosis (6 versus 10 days; *P*<0.001) and a smaller EM skin lesion diameter at the first clinical evaluation (10 versus 16 cm; *P*<0.001), and had an EM skin lesion that was more often homogeneous in appearance (65.4% versus 46.4%; *P* = 0.001), but the rate of growth in size of the EM skin lesion was similar in the two groups. There was also a trend for spirochetemic patients to more often have constitutional symptoms, thrombocytopenia and an abnormal liver function test result. In the subgroup of patients who recalled a tick bite at the site of the EM skin lesion, the mean duration of EM prior to diagnosis was shorter in the blood culture positive group than in the blood culture negative group (5 (3–8) days versus 7 (3–16) days; *P* = 0.013). More detailed information is provided in S1 Table.

Analyses using a multiple logistic regression model revealed similar associations as found using univariate analyses for proportion of patients recalling tick bite, largest diameter of EM, and homogeneous appearance of EM. Furthermore, this model also showed higher odds for the presence of constitutional symptoms (OR = 1.88, 95% CI: 1.07–3.30, *P* = 0.027) and abnormal liver enzymes (OR = 2.00, 95% CI: 1.09–3.67, *P* = 0.023) in patients with isolation of borreliae from blood in comparison to those with borreliae isolated only from skin. Detailed results are shown in Table 1.

**Table 1:** Variables related to isolation of borreliae (*Borrelia afzelii* or *Borrelia garinii*) from blood (*n* = 153) or only from skin (*n* = 153).

Pre-treatment findings	OR^a^	95% CI	*p*^b^
Tick bite	1.74	[1.01–2.98]	0.044
Largest diameter of EM (cm)	0.98	[0.95–1.00]	0.064
Homogeneous appearance of EM	1.74	[1.06–2.85]	0.028
Any constitutional symptom	1.88	[1.07–3.30]	0.027
Fever	3.01	[0.33–27.23]	0.278
Platelets < 140×10^9^/L	5.88	[0.66–52.16]	0.062
Abnormal liver enzymes	2.00	[1.09–3.67]	0.023

OR, odds ratio; CI, confidence interval.

^a^Estimated from a multiple logistic regression model with isolation of borreliae from blood as the dependent variable. Each OR is adjusted for all other variables in the table.

^b^*P values <0*.*01 were considered significant*.

Comparison of the findings in EM patients with *B*. *afzelii* spirochetemia with patients who had *B*. *afzelii* isolated only from skin (116 in each group) showed similar results to that found for spirochetemic versus non-spirochetemic patients in general. Patients with *B*. *afzelii* in blood more often recalled a tick bite at the site of EM (80/116, 69.0% versus 55/115, 48.7; *P* = 0.002) and had a shorter time interval from the bite to the onset of EM (11 versus 21 days; *P* = 0.002). Spirochetemic patients also had a shorter duration of the skin lesion prior to diagnosis (6 versus 10 days; *P* = 0.001), and had a smaller EM skin lesion at the first clinical evaluation (10 versus 15 cm; *P*<0.001) that was more often homogeneous (64.7% versus 44.8%; *P* = 0.004), but the rate of growth in size of the EM skin lesion in the two groups was similar (Table 2). Multiple logistic regression analyses (Table 3) showed significant differences between the two groups with regard to the presence of constitutional symptoms (OR = 2.42, 95% CI: 1.26–4.65, *P* = 0.006) and trends concerning the largest diameter of EM (OR = 0.95, 95% CI: 0.91–0.99, *P* = 0.011) and thrombocytopenia (OR = 8.36, 95% CI: 0.96–72.6, *P* = 0.018).

**Table 2:** Comparison of demographic, clinical, laboratory and microbiological findings according to isolation of *Borrelia afzelii* from blood or only from skin.

Pre-treatment findings	Isolation of *B*. *afzelii*		*P* value
	from blood	only from skin	
	n = 116	n = 116	
Age (years)	48.5 (35–58)	49 (36.5–58)	0.911
Male sex	51 (44.0%; 34.8–53.5%)	51 (44.0%; 34.8–53.5%)	>0.999
Tick bite ^a^	80 (69.0%; 59.7–77.2%)	55/115 (48.7%; 38.4–57.3%)	0.002
History of prior LB	9 (7.8%; 3.6–14.2%)	14 (12.1%; 6.8–19.4%)	0.380
Underlying illnesses	35 (30.2%; 22.0–39.4%)^b^	33 (28.4%; 20.5–37.6%)^c^	0.885
Incubation (days) ^d^	11 (7–16)	21 (10.5–31.5)	<0.001
Duration of EM ^e^ (days)	6 (3–14)	10 (4–30)	0.001
Location of EM			0.366
Extremities	81 (69.8%; 60.6–78.0%)	88 (75.9%; 67.0–83.3%)	
trunk	32 (27.6%; 19.7–33.7%)	28 (24.1%; 16.7–33.0%)	
head, neck	3 (2.6%; 0.5–7.4%)	0 (0; 0–3.1%)	
Largest diameter of EM (cm)	10 (7–14.5)	15.5 (12–21.5)	<0.001
Surface of EM (cm^2^) ^f^	39.15 (23.5–103.7)	122.5 (63.5–228.5)	<0.001
Spreading of EM			
Diameter ^g^ (cm/day)	1.5 (0.9–2.4)	1.2 (0.55–3.0)	0.161
Surface ^h^ (cm^2^/day)	8.2 (4.35–12.35)	8.9 (4.25–21.15)	0.247
Homogeneous appearance of EM	75 (64.7%; 55.2–73.3%)	52 (44.8%; 35.6–54.3%)	0.004
Other abnormalities on physical examination	5 (4.3%; 1.4–9.8%)	1 (0.9%; 0.0–4.7%)	0.213
Any local symptom	54 (46.6%; 37.2–56.1%)	49 (42.2%; 33.1–51.8%)	0.597
itching	43 (37.1%; 28.3–46.5%)	45 (38.8%; 29.9–48.3%)	0.892
burning	9 (7.8%; 3.6–14.2%)	10 (8.6%; 4.2–15.3%)	>0.999
pain	10 (8.6%; 4.2–15.3%)	5 (4.3%; 1.4–9.8%)	0.286
Any constitutional symptom	41 (35.3%; 27.7–44.8%)	24 (20.7%; 13.7–29.2%)	0.019
fatigue	21 (18.1%; 11.6–26.3%)	13 (11.2%; 6.1–18.4%)	0.194
headache	21 (18.1%; 11.6–26.3%)	11 (9.5%; 4.8–16.3%)	0.087
myalgia	8 (6.9%; 3.0–13.1%)	9 (7.8%; 3.6–14.2%)	>0.999
arthralgia	9 (7.8%; 3.6–14.2%)	9 (7.8%; 3.6–14.2%)	-
fever	6 (5.2%; 1.9–10.9%)	1 (0.9%; 0.0–4.7%)	0.119
dizziness	5 (4.3%; 1.4–9.8%)	1 (0.9%; 0.0–4.7%)	0.213
ESR >20 mm	3/104 (2.9%; 0.6–8.2%)	5 (4.3%; 1.4–9.8%)	0.725
WBC >10x10^9^/L	5 (4.3%; 1.4–9.8%)	4 (3.4%; 1.0–8.6%)	>0.999
WBC <4x10^9^/L	0 (0; 0–3.1%)	0 (0; 0–3.1%)	>0.999
Pts <140x10^9^/L	8 (6.9%; 3.0–13.1%)	1 (0.9%; 0.0–4.7%)	0.036
Abnormal liver enzymes	28 (24.1%; 16.7–33.0%)	19 (16.4%; 10.2–24.4%)	0.191
AST	7 (6.0%; 3.0–13.1%)	10 (8.6%; 4.2–15.3%)	0.614
ALT	15 (12.9%; 7.4–20.4%)	13 (11.2%; 6.1–18.4%)	0.840
γ-GT	18 (15.5%; 9.5–23.4%)	8 (6.9%; 3.0–13.1%)	0.061
AP	0 (0; 0–3.1%)	2 (1.7%; 0.2–6.1%)	0.498
bilirubin	9 (7.8%; 3.6–14.2%)	3 (2.6%; 0.5–7.4%)	0.138
Borrelia antibodies			
IgM	8 (6.9%; 3.0–13.1%)	12 (10.3%; 4.4–19.7%)	0.483
IgG	8 (6.9%; 3.0–13.1%)	18 (15.5%; 9.5–23.4%)	0.064
IgM and/or IgG	12 (10.3%; 5.7–17.4%)	22 (19.0%; 12.3–27.3%)	0.027
**Post-treatment findings**			
Duration of EM (days) ^i^	7 (4–17)	9 (5–16)	0.378
Treatment failure	3 (2.6%; 0.5–7.4%)	1 (0.9%; 0.0–4.7%)	0.370
NOIS	2 (1.7%; 0.2–6.1%)	0 (0%; 0.0–3.1%)	
Persistence of EM ^j^	0 (0%; 0.0–3.1%)	1 (0.9%; 0.0–4.7%)	
Persistence of borreliae in skin ^k^	1 (0.9%; 0.0–4.7%)	0 (0%; 0.0–3.1%)	

Categorical variables are summarized with frequencies and percentages and 95% confidence intervals (CI), numeric variables with medians and interquartile ranges. *P* values <0.05 were considered significant.

LB, Lyme borreliosis; EM, erythema migrans; ESR, erythrocyte sedimentation rate (normal: 0–19 mm/h; WBC, white blood cell (normal: 4–10x10^9^/L); Pts, platelets (normal: 140–340x10^9^/L); AST, aspartate aminotransferase (normal: <0.58 μkat/L); ALT, alanine aminotransferase (normal: <0.74 μkat/L); γ-GT, gamma-glutamyltransferase (normal: <0.92 μkat/L); AP, alkaline phosphatase (normal: <2.15 μkat/L); NOIS, new or increased symptoms attributed to Lyme borreliosis.

^a^ At the site of EM skin lesion.

^b^ 10 patients had arterial hypertension, 4 thyroid gland disease, 2 malignant disease, 2 diabetes, 2 musculoskeletal disease, 1 heart disease, 1 asthma, 1 liver cirrhosis, 1 schizophrenia; 11 patients had a combination of two chronic diseases.

^c^ 17 patients had arterial hypertension, 4 asthma, 2 thyroid gland disease, 2 heart disease, 2 osteoporosis, 1 psoriasis, 1 epilepsy; 4 patients had a combination of two chronic diseases.

^d^ Data for patients who recalled tick bite at the site of the skin lesion.

^e^ At enrollment.

^f^ Surface of EM was calculated using formula for ellipse surface: largest diameter x smallest diameter xπ / 4.

^g^ Largest diameter of EM at the first clinical evaluation (cm) divided by duration of EM skin lesion (days).

^h^ Surface of EM at the first clinical evaluation (cm^2^) divided by duration of EM skin lesion (days).

^i^ Information available for 152 patients in each group.

^j^ EM still visible at the visit 2–3 months after the onset of antibiotic treatment.

^k^ Isolation of borreliae from skin specimen obtained with skin rebiopsy at the site of previous erythema migrans 2–3 months after the onset of antibiotic treatment.

**Table 3:** Variables related to isolation of *Borrelia afzelii* from blood (*n* = 116) or only from skin (*n* = 116).

Pre-treatment findings	OR^a^	95% CI	*P*^b^
Tick bite	1.44	[0.77–2.71]	0.253
Duration of EM (days)	0.99	[0.98–1.01]	0.639
Largest diameter of EM (cm)	0.95	[0.91–0.99]	0.011
Homogeneous appearance of EM	1.56	[0.87–2.78]	0.134
Any constitutional symptom	2.42	[1.26–4.65]	0.006
Platelets < 140×10^9^/L	8.36	[0.96–72.6]	0.018

OR, odds ratio; CI, confidence interval.

^a^Estimated from a multiple logistic regression model with isolation of *Borrelia afzelii* from blood as the dependent variable. Each OR is adjusted for all other variables in the table.

^b^*P values* <0.01 were considered significant.

In contrast to EM caused by *B*. *afzelii*, comparison of patients with *B*. *garinii* recovered from blood versus only from skin (37 in each group) revealed no statistically significant differences in univariate analyses (S2 Table). However, in a multiple logistic regression model (S3 Table), isolation of *B*. *garinii* from blood was associated with a higher odds ratio for EM to be located on the extremities (OR = 3.27, 95% CI: 1.06–10.07, *P* = 0.032) and a higher odds ratio for the presence of abnormal liver enzymes (OR = 4.95, 95% CI: 1.36–18.06, *P* = 0.009).

Findings for the two *Borrelia* species cultured from the same source showed that in comparison to patients with isolation of *B*. *afzelii* from blood, those with a positive *B*. *garinii* blood culture (S4 Table) had significantly larger EM skin lesions (median largest diameter 20 versus 10 cm; *P*<0.001; median surface area 207 versus 39 cm^2^) that increased faster (median 2.9 cm/day versus 1.5 cm/day; *P*<0.001; 28.3 cm^2^/day versus 8.2 cm^2^/day; *P*<0.001), and more often reported itching at the site of the lesion (65% versus 37%; *P* = 0.006). A multiple logistic regression model showed that patients with *B*. *garinii* isolated from blood had higher odds for itching (OR = 3.23, 95% CI: 1.41–7.14, *P* = 0.004) but lower odds for the presence of headache (OR = 0.28, 95% CI: 0.07–1.09, *P* = 0.043) (S5 Table).

Comparison of patients with *B*. *afzelii* or *B*. *garinii* isolated from skin but not from blood revealed similar trends but less significant differences. Detailed findings are shown in S6 and S7 Tables.

### Post-treatment course and outcome

In the large majority of patients the post-treatment course was uneventful, and the outcome was favorable. Furthermore, all repeat borrelia blood cultures (147/147) were negative, while just 1/191 skin rebiopsies (136 in patients with isolation of borrelia from skin only and 55 in patients with spirochetemia) performed at the site of previous EM was borrelia culture positive.

During the 1-year follow-up period treatment failure was observed in 7/306 (2.3%) patients: 3 (1.0%) developed pronounced subjective symptoms not attributable to other causes that substantially interfered with their daily activities, 3 (1.0%) had a still visible EM skin lesion at 2–3 months after the first examination, and 1 (0.3%) had a positive follow-up skin culture 2.5 months after beginning antibiotic therapy. None of patients developed MEM or an objective extracutaneous manifestation of LB. Three of the seven patients were initially treated with doxycycline, two with cefuroxime, one with azithromycin and one with amoxicillin. All seven patients with treatment failure were re-treated with an alternative antibiotic and had a successful outcome.

Of the 7/306 patients with treatment failure, 4/153 (2.6%) had spirochetemia while 3/153 (2.0%) had a positive borrelia culture only from skin. Four treatment failures were associated with *B*. *afzelii* infection (3/116, 2.6% in patients with spirochetemia versus 1/116, 0.9% in patients with *B*. *afzelii* isolated only from skin), and three treatment failures with *B*. *garinii* infection (1/37, 2.7% in patients with spirochetemia versus 2/37, 5.4% in patients with *B*. *garinii* isolated only from skin).

No significant differences in the frequency of treatment failure were found comparing patients with isolation of borreliae from blood versus only from skin, nor according to *Borrelia* species. Furthermore, the duration of EM after beginning of antibiotic treatment was comparable in patients with isolation of borreliae from blood versus those with isolation only from skin (S1 Table) in general, as well as in the *B*. *afzelii* and *B*. *garinii* subgroups (Table 2 and S2 Table). Furthermore, no statistically significant differences in the duration of the EM skin lesion after treatment were found when comparing cases in which *B*. *afzelii* or *B*. *garinii* were isolated from blood (S4 Table) or just from skin (S6 Table).

## Discussion

Borreliae are found in the blood early in the course of LB, usually when EM is present, and only exceptionally when other manifestations of the disease are present [6, 8, 28–30]. We do not know whether in humans the spirochetemia is continuous or intermittent and how long it lasts, nor how often in untreated patients the presence of borrelia in the blood will lead to later manifestations of the disease. Published information indicates that borreliae are more often isolated from the blood of patients with MEM, a clinical consequence of dissemination, than from those with a solitary EM [9, 30]. The timing for the development of visible MEM relative to when the skin sites were seeded by hematogenous spread is unclear.

In general, the presence of other bacteria in the blood is often associated with fever and chills, and with constitutional symptoms such as malaise, fatigue, and muscle aches [31, 32]. In Europe, EM is associated with fever infrequently, mainly in children and only exceptionally in adults; typically there are no chills [3, 10–12, 14, 26, 33–38]. In the present study only 8/306 (2.6%) patients with a solitary EM reported fever. The proportion was higher in patients with borrelia isolated from blood than in those who had borreliae only recovered from skin, but the difference was not significant (7/153, 4.6% versus 1/153, 0.7%, *P* = 0.067). Furthermore, constitutional symptoms were reported more often by those with borreliae present in blood than only in skin (52/153, 34.0% versus 31/153, 20.3%, *P* = 0.010; OR in multiple regression analysis 1.88, *P* = 0.027). Nevertheless, even among patients with spirochetemia, 2/3 of patients had no systemic symptoms. However, we would like to stress that only new or increased symptoms time-related with EM, and with no other obvious explanation, qualified as constitutional (LB associated) symptoms. In a recent European study, using the same definition for LB associated symptoms, patients with MEM (the presence of multiple EM lesions indicates that hematogeneous dissemination occurred) reported LB associated constitutional symptoms more often than those with a solitary EM (93/200, 46.5% versus 96/403, 23.8%; *P*<0.001) [38]. In patients with a solitary EM the frequency of constitutional symptoms was similar (96/403, 23.8%) to that found in the present study (83/306, 27.1%), while patients with multiple EM had constitutional symptoms more often than those with solitary EM and isolation of borreliae from blood in the present study (93/200, 46.5% versus 52/153, 34.0%; *P*<0.001). Since *B*. *burgdorferi* s.l. does not produce toxins or extracellular matrix-degrading proteases, most manifestations of LB result from inflammation generated by the host immune response to the spirochete [1]. Thus, fewer symptoms, as found in the present study in patients with solitary EM and isolation of borrelia from blood, are probably associated with lower levels of inflammation in this group (at the time of spirochetemia) than in patients with MEM, which occur as sequelae of dissemination.

The findings of the current study suggest that the clinical course of solitary EM in patients with isolation of borrelia from the blood is somewhat different from that of patients with borrelia detected only in skin. Namely, patients with isolation of borreliae from blood significantly more often recalled a tick bite at the site of EM, had a shorter time interval from the bite to the onset of EM, had a shorter duration of the skin lesion prior to diagnosis, and had a smaller EM skin lesion at the time of diagnosis that was more often homogeneous. The most plausible explanation for many of these differences is that patients with spirochetemia have detected the skin lesion and come for an examination earlier due to the presence of constitutional and / or local symptoms that alert them that something is wrong. However, although both constitutional symptoms and local symptoms in this group were more common than in patients with isolation of borrelia from the skin only, these differences were not (highly) significant (34% versus 20%, *P* = 0.010; 52% versus 46%, *P* = 0.360). Since recollection of a tick bite at the site of a later skin lesion might also be a stimulus for a decision to seek medical advice earlier (due to a fear of tick-transmitted disease), we additionally assessed this possibility and found that duration of EM prior to the visit to our Outpatient LB clinic was significantly shorter in patients with a tick bite at the site of the EM than in those who did not recall tick bite (median 5 (3–12) days versus 14 (5–30) days; *P*<0.001). If the chances for spirochetemia were higher early in the course of EM and are diminishing with the duration of EM, than this would be an explanation for the higher proportion of patients recalling a tick bite at the site of the EM in the borrelia blood culture positive group.

Since *B*. *afzelii* comprised the majority (76%) of isolates, it was not a surprise that a comparison of findings in patients with isolation of *B*. *afzelii* from blood versus only from skin showed similar results to that of the combined culture positive group in terms of recollection of a tick bite, time interval from the bite to the onset of EM, duration of the skin lesion prior to diagnosis, diameter of EM at the first clinical evaluation and the proportion of patients with a homogeneous skin lesion (Table 2), while the corresponding comparisons of patients with isolation of *B*. *garinii* revealed no statistically significant differences (S2 Table). However, because the number of patients with positive blood cultures for *B*. *garinii* was much lower, the statistical power to detect differences in the comparisons made was poor. In general, the multiple regression analyses revealed similar results to the univariate analyses but also showed certain additional associations suggesting that the presence of borreliae in blood is associated with a higher frequency of constitutional symptoms, thrombocytopenia (*B*. *afzelii*) and elevated liver enzyme levels (*B*. *garinii*) than for patients with isolation of the corresponding borrelia species only from skin (Table 3 and S3 Table).

Our study also revealed that in comparison to patients with *B*. *afzelii* isolated from blood, those with *B*. *garinii* bacteremia had a larger EM skin lesion that expanded faster, more often had local symptoms (mild itching), but not systemic symptoms, and showed a trend for more frequent laboratory abnormalities including an elevated ESR, leukopenia and the presence of borrelia IgG antibodies in serum. Comparison of the findings for the skin culture positive groups showed similar but more often statistically non-significant results. These findings suggest differences in pathogenicity between the two *Borrelia* species.

The course and outcome of EM after antibiotic treatment was favorable in the large majority of patients included in the present study. The duration of EM after the initiation of antibiotic treatment was similar in patients with the isolation of borreliae from blood compared with those with isolation only from skin (S1 Table). The same was also true for the *B*. *afzelii* subgroup, as well as for the *B*. *garinii* subgroup of spirochetemic patients (Table 2 and S2 Table). Treatment failures were rare (even though the incomplete disappearance of EM 2–3 months after the beginning of antibiotic treatment was interpreted as one of the possible treatment failures, which is a rather “generous” interpretation) and did not seem to be associated with the presence of Lyme borreliae in blood or with the particular *Borrelia* species causing the infection. The proportion of patients with the post LB symptoms was very low. However, in the present study, which was restricted to patients with a single EM skin lesion, only 27% of patients (34% in spirochetemic and 20% in non-spirochetemic group) reported constitutional symptoms before treatment, i.e., a much lower proportion than in the US patients with EM. The findings of the present study are similar to that of recently published European data on adult patients with EM (16% had MEM) of whom 6% (59/977) had an incomplete response 12 months after treatment. In that study, the proportion with an incomplete response was 3.7% (10/271) in the age group 18–44 years, 6.6% (34/513) in the middle-aged group (45–64 years) and 7.8% (15/193) in patients ≥65 years old. Since the majority of patients included in the present study were young or middle aged (median age 50, IQR 36–58 years), and none had multiple EM, the 7/306 (2.3%, 95% CI: 0.9–4.7%) incomplete response is not surprising.

Our study is the first to provide data on the clinical and laboratory findings of patients with EM in whom strains of *B*. *afzelii* or *B*. *garinii* were isolated from blood. However, a larger number of patients with *B*. *garinii* infection would be needed for a more reliable assessment. Another limitation of our study is that because in univariable analyses many comparisons were made, it is highly likely that there would be some statistically significant associations by chance alone. Furthermore, although all clinical data were obtained in the same way throughout the period using a questionnaire, some approaches (e.g., serological tests to detect borrelial antibodies and blood volume for borrelia culture) changed over the 24-year study period, which is an additional limitation of the present study. Even though during whole time period the medium used for cultivation of borreliae was the same (MKP), as were cultivation technics, there was a difference in the volume of cultured plasma (until 2001 it was obtained from 5 ml of blood, from 2002 on from 9 ml of blood). However, even with larger blood volume in the second time period, the isolation rate remained low [4, 8], i.e. much lower than reported for the corresponding US patients [9], while the isolation rate of borrelia from the EM skin lesions remained comparable between our patients and US patients (≥50%). Furthermore, although the present study is purely descriptive, our results should encourage further studies to determine the clinically relevant biologic mechanisms by which different species of Lyme borrelia cause disease manifestations.

## Conclusions

In patients with a single EM spirochetemic patients differed from non-spirochetemic patients in multiple ways. Spirochetemic patients significantly more often recalled a tick bite at the site of the EM skin lesion, had a shorter time interval from the bite to the onset of EM, had a shorter duration of the skin lesion prior to diagnosis, and had a smaller EM skin lesion that was more often homogeneous in appearance. Similar results were found for the subset of spirochetemic patients infected with *B*. *afzelii* but not for those infected with *B*. *garinii*. Treatment failures were rare (7/306 patients, 2.3%) and were not associated with having spirochetemia or with which *Borrelia* species was causing the infection.

## Supporting information

S1 Table Comparison of demographic, clinical, laboratory and microbiological findings according to isolation of borreliae from blood or only from skin. (DOCX)

S2 Table Comparison of demographic, clinical, laboratory and microbiological findings according to isolation of *Borrelia garinii* from blood or only from skin. (DOCX)

S3 Table Variables related to isolation of *Borrelia garinii* from blood (n = 37) or only from skin (n = 37). (DOCX)

S4 Table Comparison of demographic, clinical, laboratory and microbiological findings according to isolation of *Borrelia afzelii* or *Borrelia garinii* from blood. (DOCX)

S5 Table Variables related to isolation of *Borrelia afzelii* (n = 116) or *Borrelia garinii* (n = 37) from blood. (DOCX)

S6 Table Comparison of demographic, clinical, laboratory and microbiological findings according to isolation of *Borrelia afzelii* or *Borrelia garinii* from skin. (DOCX)

S7 Table Variables related to isolation of *Borrelia afzelii* (n = 116) or *Borrelia garinii* (n = 37) from skin. (DOCX)
