# Supplementary material for: Characteristics of spirochetemic patients with a solitary erythema migrans skin lesion in Europe
Source: PLoS One. 2021 Apr 22;16(4):e0250198. doi: 10.1371/journal.pone.0250198 (PMC8062101; doi:10.1371/journal.pone.0250198)
Supplement: S1 Table — (DOCX) [file pone.0250198.s001.docx]

**S1 Table. Comparison of demographic, clinical, laboratory and microbiological findings according to isolation of borreliae from blood or only from skin.**

| **Pre-treatment findings** | | **Isolation from blood**  **n=153** | **Isolation only from skin**  **n=153** | ***P* value** |
| --- | --- | --- | --- | --- |
| Age (years) | | 50 (36–58) | 50 (37–59) | 0.774 |
| Male sex | | 68 (44.4%; 36.4–52.7%) | 68 (44.4%; 36.4–52.7%) | >0.999 |
| Tick bite ^a^ | | 105 (68.6%; 60.6–75.9%) | 74/152 (48.7%; 40.5–56.9%) | <0.001 |
| History of prior LB | | 13 (8.5%; 4.6–14.1%) | 20/152 (13.2%; 8.2–19.6%) | 0.260 |
| Underlying illnesses | | 47 (30.7%; 23.5–38.7%)^b^ | 39 (25.5%; 18.8–33.2%)^c^ | 0.373 |
| Incubation (days) ^d^ | | 10.5 (7–16.5) | 19 (10–30) | <0.001 |
| Duration of EM ^e^ (days) | | 6 (3–14) | 10 (4–30) | <0.001 |
| Location of EM:  extremities  trunk  head, neck | | 111 (72.5%; 64.8–79.5%)  39 (25.5%; 18.8–33.2%)  3 (2.0%; 0.4–5.6%) | 110 (71.9%; 64.1–78.9%)  43 (28.1%; 21.1–35.9%)  0 (0%; 0–2.4%) | 0.503 |
| Largest diameter of EM (cm) | | 10 (7–19) | 16 (12–22) | <0.001 |
| Surface of EM (cm^2^) ^f^ | | 51 (28–198.7) | 131 (65–240.5) | <0.001 |
| Spreading of EM  Diameter ^g^ (cm/day)  Surface ^h^ (cm^2^/day) | | 1.7 (1–3)  9.3 (4.7–18.9) | 1.5 (0.7–3.1)  11.1 (4.65–26.5) | 0.177  0.332 |
| Homogeneous appearance of EM | | 100 (65.4%; 57.3–72.9%) | 71 (46.4%; 38.3–54.6%) | 0.001 |
| Other abnormalities on physical examination | | 5 (3.3%; 1.1–7.5%) | 2 (1.3%; 0.2–4.6%) | 0.448 |
| Any local symptom  itching  burning  pain | | 80 (52.3%; 44.1–60.4%)  67 (43.8%; 35.8–52.0%)  16 (10.5%; 6.1–16.4%)  13 (8.5%; 4.6–16.1%) | 71 (46.4%; 38.3–54.6%)  61 (39.9%; 32.1–48.1%)  14 (9.2%; 5.1–14.9%)  11 (7.2%; 3.6–12.5%) | 0.360  0.562  0.848  0.832 |
| Any constitutional symptom | | 52 (34.0%; 26.5–42.1%) | 31 (20.3%; 14.2–27.5%) | 0.010 |
|  | fatigue  headache  myalgia  arthralgia  fever  dizziness | 26 (17.0%; 11.4–23.9%)  24 (15.7%; 10.3–22.4%)  8 (5.2%; 2.3–10.0%)  10 (6.5%; 3.2–11.7%)  7 (4.6%; 1.9–9.2%)  5 (3.3%; 1.1–7.5%) | 17 (11.1%; 6.6–17.2%)  13 (8.5%; 4.6–14.1%)  11(7.2%; 3.6–12.5%)  12 (8.4%; 4.1–13.3%)  1 (0.7%; 0.0–3.6%)  1 (0.7%; 0.0–3.6%) | 0.188  0.080  0.636  0.825  0.067  0.214 |
| ESR >20 mm | | 8/135 (5.9%; 2.6–11.3%) | 6 (3.9%; 1.5–8.3%) | 0.607 |
| WBC >10x10^9^/L | | 6 (3.9%; 1.5–8.3%) | 5 (3.3%; 1.1–7.5%) | >0.999 |
| WBC <4x10^9^/L | | 2 (1.3%; 0.2–4.6%) | 2 (1.3%; 0.2–4.6%) | >0.999 |
| Pts <140x10^9^/L | | 8 (5.2%; 2.3–10.0%) | 1 (0.7%; 0.0–3.6%) | 0.036 |
| Abnormal liver enzymes | | 41 (26.8%; 20.0–34.6%) | 23 (15.0%; 9.8–21.7%) | 0.017 |
|  | AST  ALT  γ-GT  AP  bilirubin | 10 (6.5%; 3.2–11.7%)  23 (15.0%; 9.8–21.7%)  23 (15.0%; 9.8–21.7%)  1 (0.7%; 0.0–3.6%)  12 (7.8%; 4.1–13.3%) | 11 (7.2%; 3.6–12.5%)  15 (9.8%; 5.6–15.7%)  10 (6.5%; 3.2–11.7%)  4 (2.6%; 0.7–6.6%)  4 (2.6%; 0.7–6.6%) | >0.999  0.225  0.027  0.371  0.072 |
| Borrelia antibodies  IgM  IgG  IgM and/or IgG | | 12 (7.8%; 4.1–13.3%)  16 (10.5%; 6.1–16.4%)  20 (13.1%; 8.2–19.5%) | 14 (9.2%; 5.1–14.9%)  24 (15.9%; 10.3–22.4%)  28 (18.3%; 12.5–25.4%) | >0.999  0.244  0.272 |
| **Post-treatment findings** | |  |  |  |
| Duration of EM (days) ^i^ | | 7 (4.5–16) | 9 (5–16) | 0.378 |
| Treatment failure  NOIS  Persistence of EM ^j^  Persistence of  borreliae in skin ^k^ | | 4 (2.6%; 0.7–6.6%)  2 (1.3%; 0.2–4.6%)  1 (0.7%; 0.0–3.6%)  1 (0.7%; 0.0–3.6%) | 3 (2.0%; 0.4–5.6%)  1 (0.7%; 0.0–3.6%)  2 (1.3%; 0.2–4.6%)  0 (0%; 0.0–2.4%) | >0.999 |

Categorical variables are summarized with frequencies and percentages and 95% confidence intervals (CI), numeric variables with medians and interquartile ranges. *P* values <0.05 were considered significant.

LB, Lyme borreliosis; EM, erythema migrans; ESR, erythrocyte sedimentation rate (normal: 0–19 mm/h; WBC, white blood cell (normal: 4–10x10^9^/L); Pts, platelets (normal: 140–340x10^9^/L); AST, aspartate aminotransferase (normal: <0.58 µkat/L); ALT, alanine aminotransferase (normal: <0.74 µkat/L); γ-GT, gamma-glutamyltransferase (normal: <0.92 µkat/L); AP, alkaline phosphatase (normal: <2.15 µkat/L); NOIS, new or increased symptoms attributed to Lyme borreliosis.

^a^ At the site of later EM skin lesion.

^b^ 11 patients had arterial hypertension, 6 thyroid gland disease, 3 malignant disease, 2 diabetes, 2 heart disease, 2 musculoskeletal disease, 2 asthma, 1 liver cirrhosis, 1 gout; 1 megaloblastic anemia, 1 glaucoma, 1 schizophrenia; 14 patients had a combination of two chronic diseases.

^c^ 19 patients had arterial hypertension, 4 asthma, 2 thyroid gland disease, 2 heart disease, 2 osteoporosis, 1 diabetes, 1 psoriasis, 1 epilepsy; 7 patients had a combination of two chronic diseases.

^d^ Data for patients who recalled tick bite at the site of the later skin lesion.

^e^At enrollment.

^f^ Surface area of EM was calculated using formula: largest diameter x smallest diameter x π / 4.

^g^ Largest diameter of EM at the first clinical evaluation (cm) divided by duration of EM skin lesion (days).

^h^ Area of EM at the first clinical evaluation (cm^2^) divided by duration of EM skin lesion (days).

^i^ Information available for 152 patients in each group.

^j^ EM still visible at the visit 2–3 months after the onset of antibiotic treatment.

^k^ Isolation of borreliae from skin specimen obtained with skin rebiopsy at the site of previous EM 2–3 months after the onset of antibiotic treatment.
