# Supplementary material for: Characteristics of spirochetemic patients with a solitary erythema migrans skin lesion in Europe
Source: PLoS One. 2021 Apr 22;16(4):e0250198. doi: 10.1371/journal.pone.0250198 (PMC8062101; doi:10.1371/journal.pone.0250198)
Supplement: S2 Table — (DOCX) [file pone.0250198.s002.docx]

**S2 Table. Comparison of demographic, clinical, laboratory and microbiological findings according to isolation of *Borrelia garinii* from blood or only from skin.**

| **Pre-treatment findings** | | **Isolation of *B. garinii*** | | ***P* value** |
| --- | --- | --- | --- | --- |
|  |  | **from blood**  **n=37** | **only from skin**  **n=37** |  |
| Age (years) | | 52 (37–59) | 53 (39–61) | 0.677 |
| Male sex | | 17 (45.9%; 29.5–63.1%) | 17 (45.9%; 29.5–63.1%) | - |
| Tick bite ^a^ | | 25 (67.6%; 50.2–82.2%) | 19 (51.4%; 34.4–68.1%) | 0.237 |
| History of prior LB | | 4 (10.8%; 3.0–25.4%) | 6/36 (16.7%; 6.4–32.8%) | 0.515 |
| Underlying illnesses | | 12 (32.4%; 18.0–49.8%)^b^ | 6 (16.2%; 6.2–32.0%)^c^ | 0.176 |
| Incubation (days) ^d^ | | 10 (7–17) | 13 (8–19) | 0.448 |
| Duration of EM ^e^ (days) | | 6 (3–12) | 7 (4–14) | 0.313 |
| Location of EM  extremities  trunk  head, neck | | 30 (81.1%; 64.8–92.0%)  7 (18.9%; 8.0–35.2%)  0 (0%; 0–9.5%) | 22 (59.5%; 42.1–75.3%)  15 (40.5%; 24.8–57.9%)  0 (0%; 0–9.5%) | 0.010 |
| Largest diameter of EM ^d^ (cm) | | 20 (12–25) | 20 (13–24) | 0.991 |
| Surface of EM (cm^2^) ^f^ | | 207 (84.8–364) | 194 (82–321.5) | 0.589 |
| Spreading of EM  Diameter ^g^ (cm/day)  Surface ^h^ (cm^2^/day) | | 2.9 (1.5–5)  28.3 (9.4–59) | 2.5 (1.3–4)  24.15 (11–40.5) | 0.384  0.651 |
| Homogeneous appearance of EM | | 25 (67.6%; 50.2–82.2%) | 19 (51.4%; 34.4–68.1%) | 0.237 |
| Other abnormalities on physical examination | | 0 (0%; 0–9.5%) | 1 (2.7%; 0.0–14.1%) | >0.999 |
| Any local symptom | | 26 (70.3%; 53.0–84.1%) | 22 (59.5%; 42.1–75.3%) | 0.465 |
| itching  burning  pain | | 24 (64.9%; 47.5–79.8%)  7 (18.9%; 8.0–35.2%)  3 (8.1%; 1.7–21.9%) | 16 (43.2%; 27.1–60.5%)  4 (10.8%; 3.0–25.4%)  6 (16.2%; 6.2–32.0%) | 0.103  0.513  0.479 |
| Any constitutional symptom | | 11 (29.7%; 15.9–47.0%) | 7 (18.9%; 8.0–35.2%) | 0.416 |
|  | fatigue  headache  myalgia  arthralgia  fever  dizziness | 5 (13.5%; 4.5–28.8%)  3 (8.1%; 1.7–21.9%)  0 (0%; 0–9.5%)  1 (2.7%; 0.0–14.1%)  1 (2.7%; 0.0–14.1%)  0 (0%; 0–9.5%) | 4 (10.8%; 3.0–25.4%)  2 (5.4%; 0.7–18.2%)  2 (5.4%; 0.7–18.2%)  3 (8.1%; 1.7–21.9%)  0 (0%; 0–9.5%)  0 (0%; 0–9.5%) | >0.999  >0.999  0.493  0.615  >0.999  - |
| ESR >20 mm | | 5/31 (16.1%; 5.5–33.7%) | 1 (2.7%; 0.0–14.1%) | 0.085 |
| WBC >10x10^9^/L | | 1 (2.7%; 0.0–14.1%) | 1 (2.7%; 0.0–14.1%) | >0.999 |
| WBC <4x10^9^/L | | 2 (5.4%; 0.7–18.2%) | 2 (5.4%; 0.7–18.2%) | >0.999 |
| Pts <140x10^9^/L | | 0 (0%; 0–9.5%) | 0 (0%; 0–9.5%) | >0.999 |
| Abnormal liver enzymes | | 13 (35.1%; 20.2–52.5%) | 4 (10.8%; 3.0–25.4%) | 0.095 |
|  | AST  ALT  γ-GT  AP  bilirubin | 3 (8.1%; 1.7–21.9%)  8 (21.6%; 9.8–38.2%)  5 (13.5%; 4.5–28.8%)  1 (2.7%; 0.0–14.1%)  3 (8.1%; 1.7–21.9%) | 1 (2.7%; 0.0–14.1%)  2 (5.4%; 0.7–18.2%)  2 (5.4%; 0.7–18.2%)  2 (5.4%; 0.7–18.2%)  1 (2.7%; 0.0–14.1%) | 0.615  0.089  0.430  >0.999  0.615 |
| Borrelia antibodies  IgM  IgG  IgM and/or IgG | | 4 (10.8%; 3.0–25.4%)  8 (21.6%; 9.8–38.2%)  8 (21.6%; 9.8–38.2%) | 2 (5.4%; 0.7–18.2%)  6 (16.2%; 6.2–32.0%)  6 (16.2%; 6.2–32.0%) | 0.674  0.766  0.766 |
| **Post-treatment findings** | |  |  |  |
| Duration of EM (days) ^i^ | | 7 (5–10) | 8 (5–10) | 0.862 |
| Treatment failure  NOIS  Persistence of EM ^j^  Persistence of  borreliae in skin ^k^ | | 1 (2.7%; 0.1–14.2%)  0 (0%; 0.0–9.5%)  1 (2.7%; 0.1–14.2%)  0 (0%; 0.0–9.5%) | 2 (5.4%; 0.7–18.2%)  1 (2.7%; 0.1–14.2%)  1 (2.7%; 0.1–14.2%)  0 (0%; 0.0–9.5%) | 0.493 |

Categorical variables are summarized with frequencies and percentages and 95% confidence intervals (CI), numeric variables with medians and interquartile ranges. *P* values <0.05 were considered significant.

LB, Lyme borreliosis; EM, erythema migrans; ESR, erythrocyte sedimentation rate (normal: 0–19 mm/h; WBC, white blood cell (normal: 4–10x10^9^/L); Pts, platelets (normal: 140–340x10^9^/L); AST, aspartate aminotransferase (normal: <0.58 µkat/L); ALT, alanine aminotransferase (normal: <0.74 µkat/L); γ-GT, gamma-glutamyltransferase (normal: <0.92 µkat/L); AP, alkaline phosphatase (normal: <2.15 µkat/L); NOIS, new or increased symptoms attributed to Lyme borreliosis.

^a^ At the site of later EM skin lesion.

^b^ 2 patients had arterial hypertension, 2 thyroid gland disease, 1 malignant disease, 1 heart disease, 1 asthma, 1 gout, 1 megaloblastic anemia, 1 glaucoma; 2 patients had a combination of two chronic diseases.

^c^ 3 patients had arterial hypertension,1 diabetes; 2 patients had a combination of two chronic diseases.

^d^ Data for patients who recalled tick bite at the site of the later skin lesion.

^e^ At enrollment.

^f^ Surface of EM was calculated using formula for ellipse surface: largest diameter x smallest diameter xπ / 4.

^g^ Largest diameter of EM at the first clinical evaluation (cm) divided by duration of EM skin lesion (days).

^h^ Surface of EM at the first clinical evaluation (cm^2^) divided by duration of EM skin lesion (days).

^i^ Information available for 152 patients in each group.

^j^ EM still visible at the visit 2–3 months after the onset of antibiotic treatment.

^k^ Isolation of borreliae from skin specimen obtained with skin rebiopsy at the site of previous EM 2–3 months after the onset of antibiotic treatment.
