# Supplementary material for: Characteristics of spirochetemic patients with a solitary erythema migrans skin lesion in Europe
Source: PLoS One. 2021 Apr 22;16(4):e0250198. doi: 10.1371/journal.pone.0250198 (PMC8062101; doi:10.1371/journal.pone.0250198)
Supplement: S3 Table — (DOCX) [file pone.0250198.s003.docx]

**S3 Table. Variables related to isolation of *Borrelia garinii* from blood (*n* = 37) or only from skin (*n* = 37).**

| **Pre-treatment findings** | **OR**^a^ | **95% CI** | ***P***^b^ value |
| --- | --- | --- | --- |
| Location of EM: Extremities | 3.27 | [1.06 – 10.07] | 0.032 |
| Abnormal liver enzymes | 4.95 | [1.36 – 18.06] | 0.009 |

OR, odds ratio; CI, confidence interval.

^a^ Estimated from a multiple logistic regression model with isolation of *Borrelia garinii* from blood as the dependent variable. Each OR is adjusted for all other variables in the table.

^b^ *P* values <0.01 were considered significant.
