# Supplementary material for: Characteristics of spirochetemic patients with a solitary erythema migrans skin lesion in Europe
Source: PLoS One. 2021 Apr 22;16(4):e0250198. doi: 10.1371/journal.pone.0250198 (PMC8062101; doi:10.1371/journal.pone.0250198)
Supplement: S4 Table — (DOCX) [file pone.0250198.s004.docx]

**S4 Table. Comparison of demographic, clinical, laboratory and microbiological findings according to isolation of *Borrelia afzelii* or *Borrelia garinii* from blood.**

| **Pre-treatment findings** | | **Isolation from blood** | | ***P* value** |
| --- | --- | --- | --- | --- |
|  |  | ***B. afzelii***  **n=116** | ***B. garinii***  **n=37** |  |
| Age (years) | | 48.5 (35–58) | 52 (37–59) | 0.556 |
| Male sex | | 51 (44.0%; 34.8–53.5%) | 17 (45.9%; 29.5–63.1%) | 0.983 |
| Tick bite ^a^ | | 80 (69.0%; 59.7–77.2%) | 25 (67.6%; 50.2–82.2%) | 0.965 |
| History of prior LB | | 9 (7.8%; 3.6–14.2%) | 4 (10.8%; 3.0–25.4%) | 0.516 |
| Underlying illnesses | | 35 (30.2%; 22.0–39.4%)^b^ | 12 (32.4%; 18.0–49.8%)^c^ | 0.956 |
| Incubation (days) ^d^ | | 11 (7–16) | 10 (7–17) | 0.906 |
| Duration of EM ^e^ (days) | | 6 (3–14) | 6 (3–12) | 0.963 |
| Location of EM:  extremities  trunk  head, neck | | 81 (69.8%; 60.6–78.0%)  32 (27.6%; 19.7–33.7%)  3 (2.6%; 0.5–7.4%) | 30 (81.1%; 64.8–92.0%)  7 (18.9%; 8.0–35.2%)  0 (0%; 0–9.5%) | 0.676 |
| Largest diameter of EM (cm) | | 10 (7–14.5) | 20 (12–25) | <0.001 |
| Surface of EM (cm^2^) ^f^ | | 39.15 (23.6–103.7) | 207 (84.8–36.4) | <0.001 |
| Spreading of EM  Diameter ^g^ (cm/day)  Surface ^h^ (cm^2^/day) | | 1.5 (0.9–2.4)  8.2 (4.35–12.35) | 2.9 (1.5–5.0)  28.3 (9.4–59.0) | 0.003  <0.001 |
| Homogenous appearance of EM | | 75 (64.7%; 55.2–73.3%) | 25 (67.6%; 50.2–82.2%) | 0.899 |
| Other abnormalities on physical examination | | 5 (4.3%; 1.4–9.8%) | 0 (0%; 0–9.5%) | 0.337 |
| Any local symptom  itching  burning  pain | | 54 (46.6%; 37.2–56.1%)  43 (37.1%; 28.3–46.5%)  9 (7.8%; 3.6–14.2%)  10 (8.6%; 4.2–15.3%) | 26 (70.3%; 53.0–84.1%)  24 (64.9%; 47.5–79.8%)  7 (18.9%; 8.0–35.2%)  3 (8.1%; 1.7–21.9%) | 0.020  0.006  0.066  >0.999 |
| Any constitutional symptom | | 41 (35.3%; 27.7–44.8%) | 11 (29.7%; 15.9–47.0%) | 0.668 |
|  | fatigue  headache  myalgia  arthralgia  fever  dizziness | 21 (18.1%; 11.6–26.3%)  21 (18.1%; 11.6–26.3%)  8 (6.9%; 3.0–13.1%)  9 (7.8%; 3.6–14.2%)  6 (5.2%; 1.9–10.9%)  5 (4.3%; 1.4–9.8%) | 5 (13.5%; 4.5–28.8%)  3 (8.1%; 1.7–21.9%)  0 (0%; 0–9.5%)  1 (2.7%; 0.0–14.1%)  1 (2.7%; 0.0–14.1%)  0 (0%; 0–9.5%) | 0.692  0.232  0.200  0.453  >0999  0.337 |
| ESR >20 mm | | 3/104 (2.9%; 0.6–8.2%) | 5/31 (16.1%; 5.5–33.7%) | 0.016 |
| WBC >10x10^9^/L | | 5 (4.3%; 1.4–9.8%) | 1 (2.7%; 0.0–14.1%) | >0.999 |
| WBC <4x10^9^/L | | 8 (6.9%; 3.0–13.1%) | 2 (5.4%; 0.7–18.2%) | 0.059 |
| Pts <140x10^9^/L | | 8 (6.9%; 3.0–13.1%) | 0 (0%; 0–9.5%) | 0.200 |
| Abnormal liver enzymes | | 28 (24.1%; 16.7–33.0%) | 13 (35.1%; 20.2–52.5%) | 0.271 |
|  | AST  ALT  γ-GT  AP  bilirubin | 7 (6.0%; 3.0–13.1%)  15 (12.9%; 7.4–20.4%)  18 (15.5%; 9.5–23.4%)  0 (0; 0–3.1%)  9 (7.8%; 3.6–14.2%) | 3 (8.1%; 1.7–21.9%)  8 (21.6%; 9.8–38.2%)  5 (13.5%; 4.5–28.8%)  1 (2.7%; 0.0–14.1%)  3 (8.1%; 1.7–21.9%) | 0.705  0.306  0.974  0.242  >0.999 |
| Borrelia antibodies  IgM  IgG  IgM and/or IgG | | 8 (6.9%; 3.0–13.1%)  8 (6.9%; 3.0–13.1%)  12 (10.3%; 5.7–17.4%) | 4 (10.8%; 3.0–25.4%)  8 (21.6%; 9.8–38.2%)  8 (21.6%; 9.8–38.2%) | 0.485  0.026  0.094 |
| **Post-treatment findings** | |  |  |  |
| Duration of EM (days) ^i^ | | 7 (4–17) | 7 (5–10) | 0.301 |
| Treatment failure  NOIS  Persistence of EM ^j^  Persistence of  borreliae in skin^k^ | | 3 (2.6%; 0.5–7.4%)  2 (1.7%; 0.2–6.1%)  0 (0%; 0.0–3.1%)  1 (0.9%; 0.0–4.7%) | 1 (2.7%; 0.1–14.2%)  0 (0%; 0.0–9.5%)  1 (2.7%; 0.1–14.2%)  0 (0%; 0.0–9.5%) | >0.999 |

Categorical variables are summarized with frequencies and percentages and 95% confidence intervals (CI), numeric variables with medians and interquartile ranges. *P* values < 0.05 were considered significant.

LB, Lyme borreliosis; EM, erythema migrans; ESR, erythrocyte sedimentation rate (normal: 0–19 mm/h; WBC, white blood cell (normal: 4–10x10^9^/L); Pts, platelets (normal: 140–340x10^9^/L); AST, aspartate aminotransferase (normal: <0.58 µkat/L); ALT, alanine aminotransferase (normal: <0.74 µkat/L); γ-GT, gamma-glutamyltransferase (normal: <0.92 µkat/L); AP, alkaline phosphatase (normal: <2.15 µkat/L); NOIS, new or increased symptoms attributed to Lyme borreliosis.

^a^ At the site of later EM skin lesion.

^b^ 9 patients had arterial hypertension, 5 thyroid gland disease, 2 malignant disease, 2 diabetes, 2 musculoskeletal disease, 1 heart disease, 1 asthma, 1 liver cirrhosis, 1 schizophrenia; 11 patients had a combination of two chronic diseases.

^c^ 2 patients had arterial hypertension, 1 asthma,1thyroid gland disease,1 heart disease,1 malignant disease, 1 gout, 1 megaloblastic anemia, 1 glaucoma; 3 patients had a combination of two chronic diseases.

^d^ Data for patients who recalled tick bite at the site of the later skin lesion.

^e^ At enrollment.

^f^ Surface of EM was calculated using formula for ellipse surface: largest diameter x smallest diameter x π / 4.

^g^ Largest diameter of EM at the first clinical evaluation (cm) divided by duration of EM skin lesion (days).

^h^ Surface of EM at the first clinical evaluation (cm^2^) divided by duration of EM skin lesion (days).

^I^ Information available for 152 patients in each group.

^j^ EM still visible at the visit 2–3 months after the onset of antibiotic treatment.

^k^ Isolation of borreliae from skin specimen obtained with skin rebiopsy at the site of previous EM 2–3 months after the onset of antibiotic treatment.
