# Supplementary material for: Characteristics of spirochetemic patients with a solitary erythema migrans skin lesion in Europe
Source: PLoS One. 2021 Apr 22;16(4):e0250198. doi: 10.1371/journal.pone.0250198 (PMC8062101; doi:10.1371/journal.pone.0250198)
Supplement: S5 Table — (DOCX) [file pone.0250198.s005.docx]

**S5 Table. Variables related to isolation of *Borrelia afzelii* (*n* = 116) or *Borrelia garinii* (*n* = 37) from blood.**

| **Pre-treatment findings** | **OR**^a^ | **95% CI** | ***P***^b^ value |
| --- | --- | --- | --- |
| Location of EM: Extremities | 0.46 | [0.17 – 1.21] | 0.102 |
| Homogenous appearance of EM | 1.15 | [0.49 – 2.71] | 0.746 |
| Itching | 0.31 | [0.14 – 0.71] | 0.004 |
| Burning | 0.38 | [0.12 – 1.18] | 0.101 |
| Headache | 3.59 | [0.92 – 14.04] | 0.043 |
| Platelets < 140×10^9^/L | 6.89 | [-0.95 – 4.82] | 0.051 |

OR, odds ratio; CI, confidence interval.

^a^ Estimated from a multiple logistic regression model with isolation of *Borrelia afzelii* from blood as the dependent variable. Each OR is adjusted for all other variables in the table.

^b^ *P* values <0.01 were considered significant.
