# Supplementary material for: Characteristics of spirochetemic patients with a solitary erythema migrans skin lesion in Europe
Source: PLoS One. 2021 Apr 22;16(4):e0250198. doi: 10.1371/journal.pone.0250198 (PMC8062101; doi:10.1371/journal.pone.0250198)
Supplement: S6 Table — (DOCX) [file pone.0250198.s006.docx]

**S6 Table. Comparison of demographic, clinical, laboratory and microbiological findings according to isolation of *Borrelia afzelii* or *Borrelia garinii* from skin.**

| **Pre-treatment findings** | | **Isolation from skin** | | ***P* value** |
| --- | --- | --- | --- | --- |
|  |  | ***B. afzelii***  **n=116** | ***B. garinii***  **n=37** |  |
| Age (years) | | 49 (36.5–58) | 53 (39–61) | 0.337 |
| Male sex | | 55 (44.0%; 34.8–53.5%) | 17 (45.9%; 29.5–63.1%) | 0.984 |
| Tick bite ^a^ | | 55/115 (48.7%; 38.4–57.3%) | 19 (51.4%; 34.4–68.1%) | 0.572 |
| History of prior LB | | 14 (12.1%; 6.8–19.4%) | 6 (16.2%; 6.2–32.0%) | 0.577 |
| Underlying illnesses | | 33 (28.4%; 20.5–37.6%)^b^ | 6 (16.2%; 6.2–32.0%)^c^ | 0.204 |
| Incubation (days) ^d^ | | 21 (10.5–31.5) | 13 (8–19) | 0.025 |
| Duration of EM ^e^ (days) | | 10 (4–30) | 7 (4–14) | 0.219 |
| Location of EM:  extremities  trunk  head, neck | | 88 (75.9%; 67.0–83.3%)  28 (24.1%; 16.7–33.0%)  0 (0%; 0–3.1%) | 22 (59.5%; 42.1–75.3%)  15 (40.5%; 24.8–57.9%)  0 (0%; 0–9.5%) | 0.001 |
| Largest diameter of EM (cm) | | 15.5 (12–21.5) | 20 (13–24) | 0.124 |
| Surface of EM (cm^2^) ^f^ | | 122.5 (63.5–228.5) | 194 (82–321.5) | 0143 |
| Spreading of EM  Diameter ^g^ (cm/day)  Surface ^h^ (cm^2^/day) | | 1.2 (0.55–3.0)  8.85 (4.25–21.15) | 2.5 (1.3–4.0)  24.15 (11.0–40.5) | 0.010  <0.001 |
| Homogenous appearance of EM | | 52 (44.8%; 35.6–54.3%) | 19 (51.4%; 34.4–68.1%) | 0.615 |
| Other abnormalities on physical examination | | 1 (0.9%; 0.0–4.7%) | 1 (2.7%; 0.0–14.1%) | 0.426 |
| Any local symptom  itching  burning  pain | | 49 (42.2%; 33.1–51.8%)  45 (38.8%; 29.9–48.3%)  10 (8.6%; 4.2–15.3%)  5 (4.3%; 1.4–9.8%) | 22 (59.5%; 42.1–75.3%)  16 (43.2%; 27.1–60.5%)  4 (10.8%; 3.0–25.4%)  6 (16.2%; 6.2–32.0%) | 0.101  0.773  0.785  0.025 |
| Any constitutional symptom | | 24 (20.7%; 13.7–29.2%) | 7 (18.9%; 8.0–35.2%) | 0.999 |
|  | fatigue  headache  myalgia  arthralgia  fever  dizziness | 13 (11.2%; 6.1–18.4%)  11 (9.5%; 4.8–16.3%)  9 (7.8%; 3.6–14.2%)  9 (7.8%; 3.6–14.2%)  1 (0.9%; 0.0–4.7%)  1 (0.9%; 0.0–4.7%) | 4 (10.8%; 3.0–25.4%)  2 (5.4%; 0.7–18.2%)  2 (5.4%; 0.7–18.2%)  3 (8.1%; 1.7–21.9%)  0 (0%; 0–9.5%)  0 (0%; 0–9.5%) | >0.999  0.735  >0.999  >0.999  >0.999  >0.999 |
| ESR (>20 mm) | | 5 (4.3%; 1.4–9.8%) | 1 (2.7%; 0.0–14.1%) | >0.999 |
| WBC >10x10^9^/L | | 4 (3.4%; 1.0–8.6%) | 1 (2.7%; 0.0–14.1%) | >0.999 |
| WBC <4x10^9^/L | | 0 (0; 0–3.1%) | 2 (5.4%; 0.7–18.2%) | 0.057 |
| Pts <140x10^9^/L | | 1 (0.9%; 0.0–4.7%) | 0 (0%; 0–9.5%) | >0.999 |
| Abnormal liver enzymes | | 19 (16.4%; 10.2–24.4%) | 4 (10.8%; 3.0–25.4%) | 0.575 |
|  | AST  ALT  γ-GT  AP  bilirubin | 10 (8.6%; 4.2–15.3%)  13 (11.2%; 6.1–18.4%)  8 (6.9%; 3.0–13.1%)  2 (1.7%; 0.2–6.1%)  3 (2.6%; 0.5–7.4%) | 1 (2.7%; 0.0–14.1%)  2 (5.4%; 0.7–18.2%)  2 (5.4%; 0.7–18.2%)  2 (5.4%; 0.7–18.2%)  1 (2.7%; 0.0–14.1%) | 0.298  0.525  >0.999  0.247  >0.999 |
| Borrelia antibodies  IgM  IgG  IgM and/or IgG | | 12 (10.3%; 4.4–19.7%)  18 (15.5%; 9.5–23.4%)  22 (19.0%; 12.3–27.3%) | 2 (5.4%; 0.7–18.2%)  6 (16.2%; 6.2–32.0%)  6 (16.2%; 6.2–32.0%) | 0.520  0.875  0.369 |
| **Post-treatment findings** | |  |  |  |
| Duration of EM (days) ^i^ | | 9 (5–16) | 8 (5–10) | 0.100 |
| Treatment failure  NOIS  Persistence of EM ^j^  Persistence of  borreliae in skin ^k^ | | 1 (0.9%; 0.0–4.7%)  0 (0%; 0.0–3.1%)  1 (0.9%; 0.0–4.7%)  0 (0%; 0.0–3.1%) | 2 (5.4%; 0.7–18.2%)  1 (2.7%; 0.1–14.2%)  1 (2.7%; 0.1–14.2%)  0 (0%; 0.0–9.5%) | 0.145 |

Categorical variables are summarized with frequencies and percentages and 95% confidence intervals (CI), numeric variables with medians and interquartile ranges. *P* values < 0.05 were considered significant. LB, Lyme borreliosis; EM, erythema migrans; ESR, erythrocyte sedimentation rate (normal: 0–19 mm/h); WBC, white blood cell (normal: 4–10x10^9^/L); Pts, platelets (normal: 140–340x10^9^/L); AST, aspartate aminotransferase (normal: <0.58 µkat/L); ALT, alanine aminotransferase (normal: <0.74 µkat/L); γ-GT, gamma-glutamyltransferase (normal: <0.92 µkat/L); AP, alkaline phosphatase (normal: <2.15 µkat/L); NOIS, new or increased symptoms attributed to Lyme borreliosis.

^a^ At the site of later EM skin lesion.

^b^ 17 patients had arterial hypertension, 1 diabetes, 2 thyroid gland disease, 2 heart disease, 2 musculoskeletal disease, 5 asthma; 4 patients had a combination of two chronic diseases.

^c^ 3 patients had arterial hypertension,1 diabetes; 2 patients had a combination of two chronic diseases.

^d^ Data for patients who recalled tick bite at the site of the later skin lesion.

^e^ At enrollment.

^f^ Surface of EM was calculated using formula for ellipse surface: largest diameter x smallest diameter x π / 4.

^g^ Largest diameter of EM at the first clinical evaluation (cm) divided by duration of EM skin lesion (days).

^h^ Surface of EM at the first clinical evaluation (cm^2^) divided by duration of EM skin lesion (days).

^i^ Information available for 152 patients in each group.

^j^ EM still visible at the visit 2–3 months after the onset of antibiotic treatment.

^k^ Isolation of borreliae from skin specimen obtained with skin rebiopsy at the site of previous EM 2–3 months after the onset of antibiotic treatment.
