# Supplementary material for: Characteristics of spirochetemic patients with a solitary erythema migrans skin lesion in Europe
Source: PLoS One. 2021 Apr 22;16(4):e0250198. doi: 10.1371/journal.pone.0250198 (PMC8062101; doi:10.1371/journal.pone.0250198)
Supplement: S7 Table — (DOCX) [file pone.0250198.s007.docx]

**S7 Table. Variables related to isolation of *Borrelia afzelii* (*n* = 116) or *Borrelia garinii* (*n* = 37) from skin.**

| **Pre-treatment findings** | **OR**^a^ | **95% CI** | ***P***^b^ value |
| --- | --- | --- | --- |
| Underlying illnesses | 1.92 | [0.72 – 5.13] | 0.175 |
| Homogenous appearance of EM | 0.80 | [0.37 – 1.75] | 0.575 |
| Pain | 0.28 | [0.08 – 1.00] | 0.052 |
| Abnormal liver enzymes | 1.62 | [0.50 – 5.30] | 0.407 |

OR, odds ratio; CI, confidence interval.

^a^ Estimated from a multiple logistic regression model with isolation of *Borrelia afzelii* from skin as the dependent variable. Each OR is adjusted for all other variables in the table.

^b^ *P* values <0.01 were considered significant.
